# Supplementary material for: Splice-Junction-Based Mapping of Alternative Isoforms in the Human Proteome
Source: Cell Rep. Author manuscript; Available in PMC 2020 Jan 15. (PMC6961840; doi:10.1016/j.celrep.2019.11.026)
Supplement: 3 [file NIHMS1546469-supplement-3.zip › DF2/PXD006675/MitralValve-81-Q5VST9-TVAELAVQAPR.pdf]

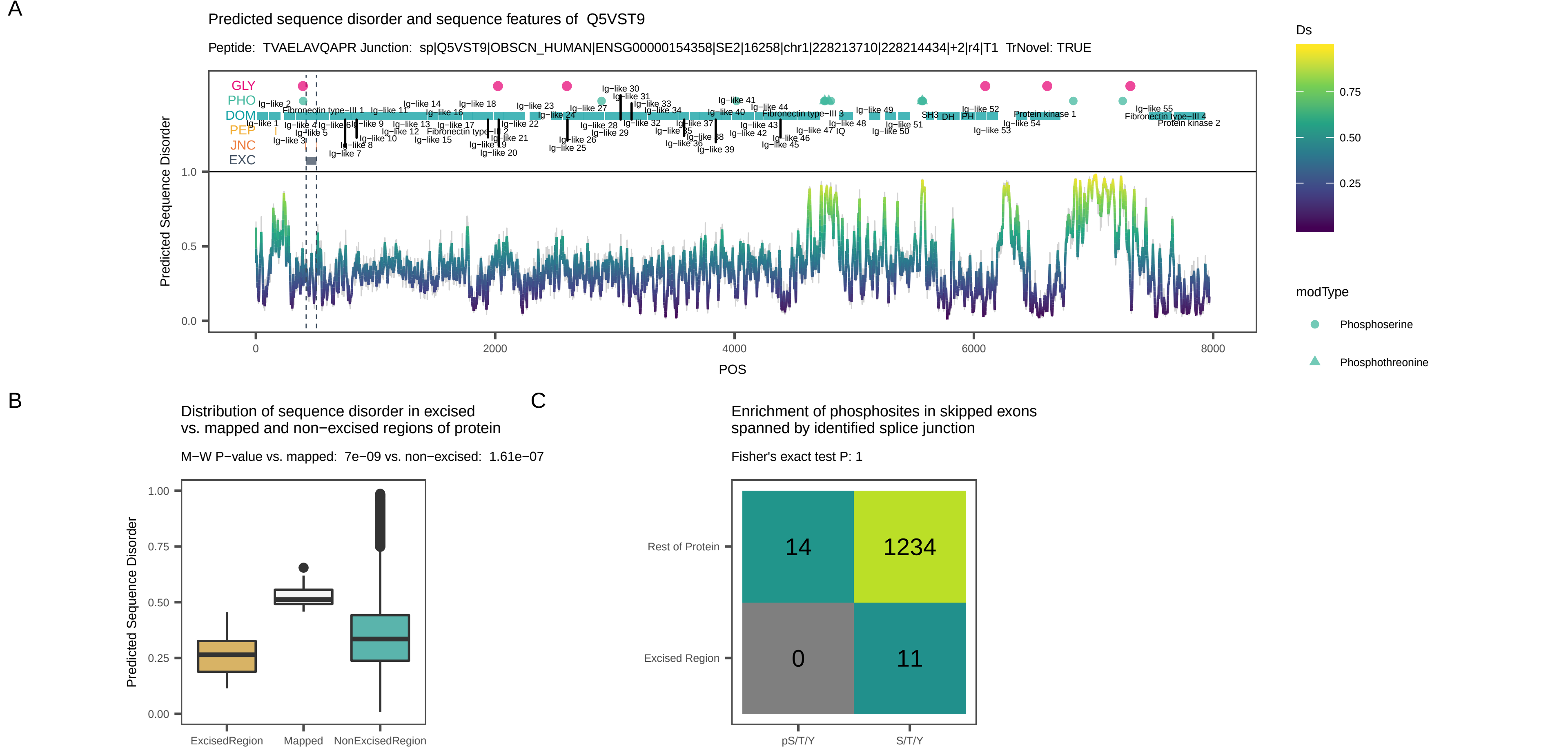
A
Predicted sequence disorder and sequence features of Q5VST9
Peptide: TVAELAVQAPR Junction: sp|Q5VST9|OBSCN_HUMAN|ENSG00000154358|SE2|16258|chr1|228213710|228214434|+2|r4|T1 TrNovel: TRUE
GLY
PHO
DOM
PEP
JNC
EXC
Predicted Sequence Disorder
POS
Ds
modType
Phosphoserine
Phosphothreonine
B
Distribution of sequence disorder in excised vs. mapped and non−excised regions of protein
M−W P−value vs. mapped: 7e−09 vs. non−excised: 1.61e−07
Predicted Sequence Disorder
ExcisedRegion
Mapped
NonExcisedRegion
C
Enrichment of phosphosites in skipped exons spanned by identified splice junction
Fisher's exact test P: 1
Rest of Protein
Excised Region
14
1234
0
11
pS/T/Y
S/T/Y
